# Postpartum Depression – From Evolutionary Concepts to Neuropsychoendocrinological Backgrounds and New Treatment Medications

**DOI:** 10.31083/AP50183

**Published:** 2026-09-07

**Authors:** Dena Sadeghi-Bahmani, Thorsten Mikoteit, Serge Brand

**Affiliations:** ^1^Department of Clinical Psychology, The Wright Institute, Berkeley, CA 94704, USA; ^2^Department of Psychology, Stanford University, Stanford, CA 94305, USA; ^3^Department of Epidemiology and Population Health, Stanford University, Stanford, CA 94305, USA; ^4^Center for Affective, Stress and Sleep Disorders, Psychiatric University of Basel, 4002 Basel, Switzerland; ^5^Faculty of Medicine, University of Basel, 4056 Basel, Switzerland; ^6^Private Clinic of Psychiatry, Psychotherapy and Psychosomatics, 67246 Dirmstein, Germany; ^7^Department of Applied Psychology, Kalaidos Private University of Applied Sciences, 8050 Zurich, Switzerland; ^8^Health Institute, Substance Abuse Prevention Research Center, Department of Psychiatry, Kermanshah University of Medical Sciences (KUMS), 6714686698 Kermanshah, Iran; ^9^Center for Disaster Psychiatry and Disaster Psychology; Center of Competence of Disaster Medicine of the Swiss Armed Forces, 4002 Basel, Switzerland

Both the bipedal locomotion about 4.4 million years ago, including the decreased pelvic circumference and narrowed vaginal tract, and the rapid brain expansion about 500,000 years ago, led to the so-called obstetric dilemma [1]. With this, both pregnancy and childbirth have become strenuous and risky [2]. As a result, and to cope with such strain, a few days after delivery, 40%–80% of mothers suffer from symptoms of depression (“baby blues”) [3], and about 17% [4] to 20% of mothers are suffering from postpartum depression (PPD) in the first six months following delivery [3,5,6,7,8,9]. Importantly, within the context of evolutionary psychology and psychiatry, a depressive phase is considered an adaptive process to preserve energy, to withdraw from challenging social interactions, and to enhance the efforts and resources of the social environment [10,11,12]. Typically, females with PPD report a wide variety of symptoms such as sad and depressed mood, emotional tension, agitation, irritability, social and interactional withdrawal, feelings of worthlessness and guilt, severe sleep disturbances, and signs of suicidal behavior [3,13]. Likewise, mothers with PPD might show less responsivity and sensitivity to the newborn’s behavior and interactional efforts [3]. Given this, PPD fulfills the criteria of a major depressive disorder, with the onset within four weeks after delivery [13].

To explain the emergence and maintenance of PPD, in the present Editorial, we focus on (1) evolutionary psychology and psychiatry, and (2) neuroendocrinological research, including (3) two novel medication options.

## 1. Evolutionary Psychology and Psychiatry and Postpartum Depression (PPD) 

Concepts of evolutionary psychiatry have gained increased scientific attention [14]. From the viewpoint of evolutionary psychology [9,12,15,16,17,18] and evolutionary psychiatry [10], in ancestral times, PPD is considered the young mother’s strategy to promote the reallocation of limited resources after a physically and psychologically demanding pregnancy and childbirth to self-preservation [10]. More specifically, and considering the evolutionary context of small-scale societies of about 120–150 individuals with kin-based organizations [19], the mother’s withdrawal from the social and economic activities implies high costs on the other group members. By contrast, the manifestation of symptoms of clinical depression is also an honest and credible signal to the kin members that cooperation is currently not possible. Furthermore, other females (the allomaternal support shared caregiving of sisters, grandmothers, aunts, or female cousins) of such kin-based societies could care for the newborn, including breastfeeding. Given this context, from the viewpoint of evolutionary psychology and psychiatry, PPD is considered an explicitly favorable adaptation to the constraints of survival. However, the contrast between evolutionary adaptive processes and our current lifestyle mirrors what is called evolutionary mismatch [20]. Given this, the lack of social and partner support [12,15,16,21,22] as a psychosocial risk factor for PPD further substantiates the evolutionary mechanisms of PPD. To support this assumption, the prevalence of postpartum depression or distress is much lower in today’s typical hunter-gatherer societies, such as the Hadza in Tanzania [23]. Regarding the development of the newborn, it is plausible to assume that in ancient times, above all, the allomaternal support and shared caregiving provided the newborn with breastfeeding, warmth, and calming, responsive behavior. By contrast, and taking into consideration the evolutionary mismatch and current lifestyle behavior mostly characterized by a two-parent family system, there is evidence that the mother’s postpartum depression is associated with an unfavorable mother-child interaction (see [24] for a systematic review and meta-analysis). Dou et al. [25] observed among 686 mother-child pairs that higher scores for mothers’ postpartum depression were associated with lower scores for infants’ communication styles, gross- and fine-motor skills, prosocial behavior, and overall cognitive, emotional, behavioral, and interactional development at the age of 12 months. Overall, there is sufficient evidence that today’s mothers’ postpartum depression is associated with more cognitive, emotional, behavioral, and social/interactional developmental issues of the offspring.

## 2. Results From Neuroendocrinological Research

Physiologically, there is a gradual rise of steroid hormones during pregnancy and a steep decline in postpartum. However, trajectories of hormonal adaptations seem to differ in females who develop PPD: Prenatal lower cortisol concentrations predicted the odds of suffering from PPD, though studies vary regarding assessment methods, time points, and lab analysis [26]. The dynamics of the hypocortisolism-PPD link are very similar to what has been observed among individuals suffering from post-traumatic stress disorder (PTSD) [27], where the organism drops to a state of generalized psychophysiological dysfunctionality. To illustrate this concept, Sadeghi-Bahmani et al. [28] assessed 129 mothers three days and 12 weeks after delivery. Participants provided hair strands for the analysis of hair glucocorticoid concentrations 12 weeks before and 12 weeks after delivery. Pre- and postpartum lower hair cortisol in relation to hair cortisol correlated with exposure to prenatal stress and higher PPD symptom scores. Jahangard et al. [29] assessed 48 females with PPD and 50 healthy controls. All participants provided hair strands to assess hair steroids (e.g., cortisol, cortisone, progesterone) 12 weeks before and 12 weeks after delivery. Compared to females without PPD, females with PPD had statistically significantly lower cortisol, cortisone, and progesterone concentrations both during the last trimester and 12 weeks after delivery. In addition, lower prenatal hair cortisone and lower prenatal hair progesterone levels predicted higher depression scores 12 weeks after delivery. Finally, there is accumulating evidence that low concentrations of progesterone and allopregnanolone (the latter is a metabolite of progesterone) are associated with higher scores for anxiety and depression during postpartum [30].

## 3. Two Novel Medications

The observation of significantly lower progesterone concentrations [30], along with allopregnanolone, in females with PPD has led to the formulation of two novel medications:

First, Brexanolone is chemically identical to allopregnanolone. It is a positive allosteric modulator of the GABA-A receptor and decreases symptoms of anxiety and depression. Brexanolone is administered intravenously for 60 consecutive hours, and symptoms of PPD decreased after 2.5 days and remained consistently low over 30 days [31,32,33].

Second, Zuranolone is an orally administered neuroactive steroid, which is a synthetic compound directly derived from allopregnanolone, and which also enhances the inhibitory effects of GABA receptors, leading to decreased symptoms of anxiety and depression. Beneficial effects are observed as early as the third day after administration, and the decreased levels of depression remain stable over 40 days [34,35,36].

In our opinion, both concepts of evolutionary psychology and psychiatry and neuroendocrinology complement each other and offer a comprehensive explanation of the emergence, maintenance, and treatment of postpartum depression. Novel pharmacological treatment opportunities offer the possibility to treat PPD more effectively (see Fig. 1). On a practical and behavioral level, concepts of evolutionary psychiatry and psychology suggest that the young mother should establish a broad, solid, and trusting social network of relatives and peers from the very beginning of pregnancy. The consequences of low levels of progesterone, allopregnanolone, and cortisone imply that women are more vulnerable to stress with symptoms of anxiety, while their hypothalamic–pituitary–adrenal (HPA) axis is still inflexible to functionally cope with stress challenges. This is in line with the above-mentioned hypothesis that vulnerable postpartum females depend on a protective and nursing social environment. Complementarily, the two novel allopregnanolone-like medications Brexanolone and Zuranolone might ameliorate the (patho) physiologic gap between the challenge of pregnancy and labor, and the cognitive-emotional and behavioral capability during the early postpartum period.

**Fig. 1.**
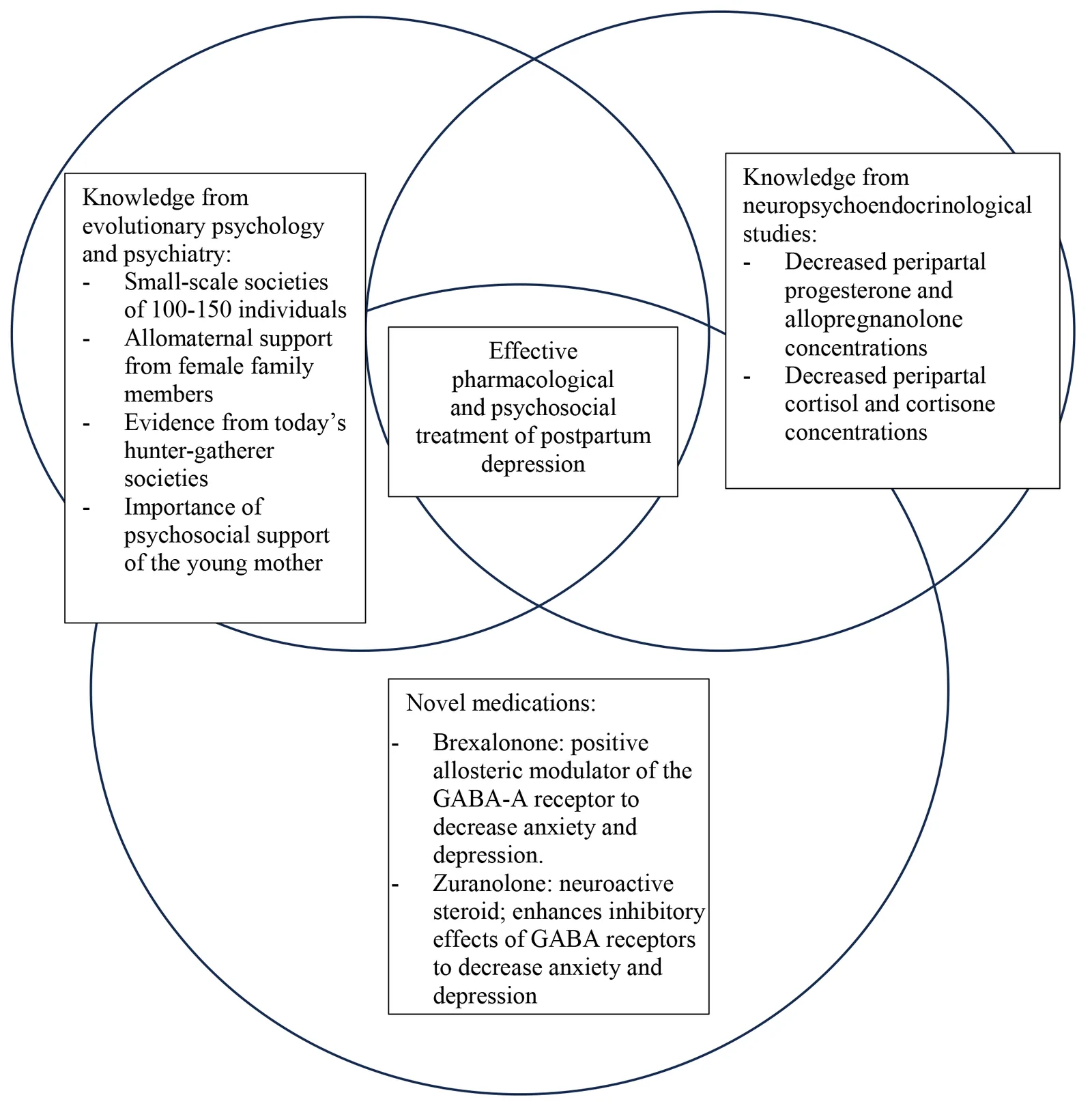
**Three dimensions to explain the emergence and dynamics of postpartum depression from the view point of evolutionary psychiatry and psychology, from neuroendocrinological research, and from novel medication treatments**.

## References

[1] Pavličev M, Romero R, Mitteroecker P (2020). Evolution of the human pelvis and obstructed labor: new explanations of an old obstetrical dilemma. *American Journal of Obstetrics and Gynecology*.

[2] Buss DM (2024). Evolutionary Psychology. *The new science of the mind*.

[3] Moreira LKS, Moreira CVL, Custódio CHX, Dias MLP, Rosa DA, Ferreira-Neto ML (2023). Post-partum depression: From clinical understanding to preclinical assessments. *Frontiers in Psychiatry*.

[4] Wang Z, Liu J, Shuai H, Cai Z, Fu X, Liu Y (2021). Mapping global prevalence of depression among postpartum women. *Translational Psychiatry*.

[5] O’Hara MW, McCabe JE (2013). Postpartum depression: current status and future directions. *Annual Review of Clinical Psychology*.

[6] O’Hara MW, Wisner KL (2014). Perinatal mental illness: definition, description and aetiology. *Best Practice & Research. Clinical Obstetrics & Gynaecology*.

[7] Brummelte S, Galea LAM (2016). Postpartum depression: Etiology, treatment and consequences for maternal care. *Hormones and Behavior*.

[8] Hahn-Holbrook J, Cornwell-Hinrichs T, Anaya I (2018). Economic and Health Predictors of National Postpartum Depression Prevalence: A Systematic Review, Meta-analysis, and Meta-Regression of 291 Studies from 56 Countries. *Frontiers in Psychiatry*.

[9] Hahn-Holbrook J, Haselton M (2014). Is Postpartum Depression a Disease of Modern Civilization?. *Current Directions in Psychological Science*.

[10] Del Giudice M (2018). *Evolutionary Psychopathology - A Unified Approach*.

[11] Brüne M (2015). Textbook of evolutionary psychiatry and psychosomatic medicine. The origins of psychopathology.

[12] Hagen EH (2011). Evolutionary theories of depression: a critical review. *Canadian Journal of Psychiatry. Revue Canadienne De Psychiatrie*.

[13] American Psychiatric Association (2022). Diagnostic and statistical manual of mental disorders (DSM-5 TR).

[14] Beyer C, Robinson C, Stein DJ (2025). Highly Cited Articles in Evolutionary Psychiatry: Assessment With a Quality and Error Rating Scale. *Alpha Psychiatry*.

[15] Hagen EH (1999). The Functions of Postpartum Depression. *Evolution and Human Behavior*.

[16] Hagen EH (2002). Depression as bargaining: The case postpartum. *Evolution and Human Behavior*.

[17] Hagen EH, Thomson JA (2004). Social navigation hypothesis of depression revisited. *Journal of Affective Disorders*.

[18] Myers S, Burger O, Johns SE (2016). Postnatal depression and reproductive success in modern, low-fertility contexts. *Evolution, Medicine, and Public Health*.

[19] Dunbar RIM (1998). The social brain hypothesis. *Evolutionary Anthropology: Issues, News, and Reviews*.

[20] Hoogland M, Ploeger A (2022). Two Different Mismatches: Integrating the Developmental and the Evolutionary-Mismatch Hypothesis. *Perspectives on Psychological Science: a Journal of the Association for Psychological Science*.

[21] Zhao XH, Zhang ZH (2020). Risk factors for postpartum depression: An evidence-based systematic review of systematic reviews and meta-analyses. *Asian Journal of Psychiatry*.

[22] Modde Epstein C, Rice MJ, French JA, Kupzyk KA, Houfek JF (2024). Social Support Buffers the Effects of Prenatal Depressed Mood: A Mixed-Methods Study. *Journal of the American Psychiatric Nurses Association*.

[23] Chaudhary N, Salali GD, Abed R, St John-Smith P (2022). Hunter-Gatherers, Mismatch and Mental Disorder. *Evolutionary Psychiatry: Current Perspectives on Evolution and Mental Health*.

[24] O’Dea GA, Youssef GJ, Hagg LJ, Francis LM, Spry EA, Rossen L (2023). Associations between maternal psychological distress and mother-infant bonding: a systematic review and meta-analysis. *Archives of Women’s Mental Health*.

[25] Dou L, Zhang X, Lei L, Ye Y, Sun S, Huang Z (2025). Maternal Perinatal Depression and Infant Behavioural Development: A Potential Cumulative Effect. *Stress and Health: Journal of the International Society for the Investigation of Stress*.

[26] Hill KE, Cárdenas EF, Yu E, Hammond R, Humphreys KL, Kujawa A (2025). A systematic review of associations between hormone levels in hair and peripartum depression. *Psychoneuroendocrinology*.

[27] Yehuda R (2002). Current status of cortisol findings in post-traumatic stress disorder. *The Psychiatric Clinics of North America*.

[28] Sadeghi-Bahmani D, Brand S, Meinlschmidt G, Tegethoff M, Kurath J, Bürki N (2025). Prepartal Stress, Prepartal and Postpartal Hair Glucocorticoid Concentrations, and Symptoms of Postpartum Depression 3 Days and 12 Weeks After Delivery. *Biological Psychiatry Global Open Science*.

[29] Jahangard L, Mikoteit T, Bahiraei S, Zamanibonab M, Haghighi M, Sadeghi Bahmani D (2019). Prenatal and Postnatal Hair Steroid Levels Predict Post-Partum Depression 12 Weeks after Delivery. *Journal of Clinical Medicine*.

[30] Sundström-Poromaa I, Comasco E, Sumner R, Luders E (2020). Progesterone - Friend or foe?. *Frontiers in Neuroendocrinology*.

[31] Edinoff AN, Odisho AS, Lewis K, Kaskas A, Hunt G, Cornett EM (2021). Brexanolone, a GABAA Modulator, in the Treatment of Postpartum Depression in Adults: A Comprehensive Review. *Frontiers in Psychiatry*.

[32] Patatanian E, Nguyen DR (2022). Brexanolone: A Novel Drug for the Treatment of Postpartum Depression. *Journal of Pharmacy Practice*.

[33] Zheng W, Cai DB, Zheng W, Sim K, Ungvari GS, Peng XJ (2019). Brexanolone for postpartum depression: A meta-analysis of randomized controlled studies. *Psychiatry Research*.

[34] Deligiannidis KM, Meltzer-Brody S, Gunduz-Bruce H, Doherty J, Jonas J, Li S (2021). Effect of Zuranolone vs Placebo in Postpartum Depression: A Randomized Clinical Trial. *JAMA Psychiatry*.

[35] Deligiannidis KM, Meltzer-Brody S, Maximos B, Peeper EQ, Freeman M, Lasser R (2023). Zuranolone for the Treatment of Postpartum Depression. *The American Journal of Psychiatry*.

[36] Sharma R, Bansal P, Saini L, Sharma N, Dhingra R (2024). Zuranolone, a neuroactive drug, used in the treatment of postpartum depression by modulation of GABAA receptors. *Pharmacology, Biochemistry, and Behavior*.

